# The role of alcohol in the management of hypertension in patients in European primary health care practices – a survey in the largest European Union countries

**DOI:** 10.1186/s12875-016-0529-5

**Published:** 2016-09-08

**Authors:** Jürgen Rehm, Jose Angel Arbesu Prieto, Markus Beier, Didier Duhot, Alessandro Rossi, Bernd Schulte, José Zarco, Henri-Jean Aubin, Michael Bachmann, Carsten Grimm, Ludwig Kraus, Jakob Manthey, Emanuele Scafato, Antoni Gual

**Affiliations:** 1 Social and Epidemiological Research Department, Centre for Addiction and Mental Health, Toronto, Canada; 2 Addiction Policy, Dalla Lana School of Public Health, University of Toronto, Toronto, Canada; 3 Faculty of Medicine, Medical Sciences Building, Institute of Medical Science, University of Toronto, Toronto, Canada; 4 Department of Psychiatry, University of Toronto, Toronto, Canada; 5 Institute of Clinical Psychology and Psychotherapy & Center of Clinical Epidemiology and Longitudinal Studies (CELOS), Technische Universität Dresden, Dresden, Germany; 6 WHO Collaborating Center for Mental Health and Addiction, Centre for Addiction and Mental Health, Toronto, Canada; 7 Primary Health Care Center La Eria Oviedo, Oviedo, Spain; 8 Primary Care Spanish Society SEMERGEN, Madrid, Spain; 9 Bavarian GP association (BHÄV), Munich, Germany; 10 Société Française de Médecine Générale, Issy les Moulineaux, France; 11 DUMG SMBH Université Paris 13, Bobigny, France; 12 CMS Cornet, Pantin, France; 13 Italian College of General Practitioners, Florence, Italy; 14 Centre for Interdisciplinary Addiction Research, Hamburg University, Universitätsklinik Hamburg-Eppendorf, Hamburg, Germany; 15 Primary Health Care Center Ibiza, Servicio Madrileño de Salud, Madrid, Spain; 16 Sociedad Española de Medicina Familiar y Comunitaria (semFYC), Madrid, Spain; 17 Departamento Medicina Interna, Universidad Complutense de Madrid, Madrid, Spain; 18 Université Paris-Saclay, Univ. Paris-Sud, UVSQ, CESP, INSERM, Villejuif, France; 19 APHP, Hôpitaux Universitaires Paris-Sud, Villejuif, France; 20 Copentown Healthcare Consultants, Cape Town, South Africa; 21 General Practitioner, Bradford, UK; 22 Royal College of General Practitioners, London, UK; 23 IFT Institut für Therapieforschung, Munich, Germany; 24 Centre for Social Research on Alcohol and Drugs (SoRAD), Stockholm University, Stockholm, Sweden; 25 WHO Collaborating Center for Health Promotion and Research on Alcohol and Alcohol-related Health Problems, Rome, Italy; 26 Population Health Unit, National Observatory on Alcohol, CNESPS, Rome, Italy; 27 Società Italiana di Alcologia (SIA), Italian Society of Alcohology, Bologna, Italy; 28 Addictions Unit, Psychiatry Department, Neurosciences Institute, Hospital Clinic, Barcelona, Spain; 29 Institut d’Investigacions Biomèdiques August Pi i Sunyer (IDIBAPS), Barcelona, Spain; 30 Red de Trastornos Adictivos (RTA - RETICS), Instituto de Salud Carlos III, Barcelona, Spain; 31 Technische Universität Dresden, Chemnitzer Str. 46, 01187 Dresden, Germany

**Keywords:** Primary health care, Blood pressure, Hypertension, Hazardous drinking, Alcohol use disorders, Disease management, Screening

## Abstract

**Background:**

Even though addressing lifestyle problems is a major recommendation in most guidelines for the treatment of hypertension (HTN), alcohol problems are not routinely addressed in the management of hypertension in primary health care.

**Methods:**

Internet based survey of 3081 primary care physicians, recruited via the mailing lists of associations for general practitioners (GPs) in France, Germany, Italy, Spain and the UK. Clinical practice, attitudes, knowledge, education and training were assessed. Logistic regression to predict screening, brief intervention and treatment for alcohol dependence in the management of hypertension were assessed.

**Results:**

Overall, about one third of the interviewed GPs reported sufficient screening in cases with HTN (34.0 %, 95 % confidence interval (CI):32.1–35.8 %). One out of five GPs screened and delivered brief interventions in HTN patients with hazardous consumption (22.2 %, 95 % CI: 20.6–23.8 %) and about one in 13 GPs provided treatment for HTN patients with alcohol dependence other than advice or brief intervention (7.8 %, 95 % CI: 6.8–8.9 %). Post-graduate training and belief in their effectiveness predicted interventions. There were marked differences between countries.

**Conclusions:**

While current interventions were overall low, marked differences between countries indicate that current practices could be improved. Education and post-graduate training seems to be key in improving clinical practice of including interventions for problematic alcohol consumption and alcohol dependence in primary health care.

**Electronic supplementary material:**

The online version of this article (doi:10.1186/s12875-016-0529-5) contains supplementary material, which is available to authorized users.

## Background

Hypertension (HTN) is the single most important risk factor for mortality and burden of disease, globally and especially for high income countries in Europe [1]. Its main effect is on cardiovascular outcomes, and consequently, reduction of blood pressure is among the risk factor targets of the World Health Organisation ‘Global Action Plan for Prevention and Control of Non-communicable Diseases’ for the period 2013–2020 [2]. Primary health care has traditionally had a key role in the detection and the management of HTN [3]. Part of this management involves advice and interventions on lifestyle factors underlying HTN, and guidelines recommend lifestyle changes as important means to reduce blood pressure, prevent and/or avoid medication for HTN [3–5]. Both epidemiology and randomized trials converge in demonstrating that alcohol consumption, in particular heavy drinking, is one of the most important lifestyle based risk factors for HTN [6–9].

However, the mortality and disease burden attributable to HTN has increased globally since 1990 [1] and large European surveys still show a large proportion of adults with uncontrolled HTN (http://apps.who.int/gho/data/?theme=home), indicating the need for further action. Of all lifestyle factors, alcohol seems to be the least intervened in the management of HTN [10–13], which is no surprise given the low screening and intervention rates for hazardous drinking and alcohol use disorder in primary health care [14, 15]. Interventions for hazardous drinking are scarce [15–17]; and alcohol use disorders have the lowest treatment rate of all mental disorders [18–20], despite evidence that there are effective interventions available for both hazardous drinking and for alcohol use disorders [21, 22], which could be implemented at the primary care level [23, 24].

Thus, improving alcohol interventions in primary health care promises to yield substantial health benefits [10–13, 25]. The main question to realize this potential is how to best implement such interventions [23], both for hazardous drinking and for alcohol use disorders, as part of routine management of HTN. Together with primary care associations in the five largest countries in the European Union (France, Germany, Italy, Spain, and the United Kingdom (UK)), we developed a survey of general practitioners (GPs) to explore knowledge, attitudes and clinical practice of lifestyle interventions in the management of HTN and to help a potential implementation of alcohol interventions (Baseline Alcohol Screening and Intervention Survey (BASIS)).

## Methods

### Design of the BASIS survey and pilot

All authors were involved in drafting and finalizing the survey, originally in English. After an empirical pilot study in five countries (*N* = 41 respondents), the survey was translated into French, German, Italian, and Spanish and the national versions were again tested and finalized with the help of local experts. A brief summary of the survey and its subsections are given in Additional file 1. It contained 28 core items (in addition to a few country-specific items) and was put online in all languages using SurveyMonkey (http://www.surveymonkey.com). The English version of the survey can be found online (Additional file 2). The theoretical basis was the Information-Motivation-Behavioural Skills model [26, 27], which stipulates that information and education is not sufficient to adopt behaviours; in addition there needs to be motivation and behavioural skills. This model had been adopted to care of non-communicable diseases [28].

### Survey implementation

In each of the five countries, regional or nationwide GP associations disseminated the web link to the survey to their members, mainly via electronic mail (for details see Table 1). The median completion time was 8.8 min, with a span from under 2 min to over an hour). Four responses were removed from the data set due to suspicion of being duplicates. The entire survey was answered by 2468 respondents (80.1 % of those who started: 3081) between September 29 and December 1, 2015.

**Table 1:** Assessment details by country

Country	Region of drawn sample	Local responsibles	Incentives	Response rate^a^	Number of complete responses
France	National sample	SFMG	None	8.5 %	512
Germany	Mostly Bavaria^b^	BHÄV	No personal incentives; €15 paid to BHÄV suborganisation	2.3 %	103
	Hamburg	CIAR	€15 voucher	7.9 %	88
Italy	National sample	SIMG	None	10.1 %	360
Spain	National sample	semFYC	None	9.4 %	802
	National sample	semergen	None	1.1 %	95
UK	National sample	MediConf	£10 voucher	4.1 %	508
Total				5.7 %	2468

Note. *SFMG* Société Française de Médecine Générale, *BHÄV* Bayerischer Hausärzteverband, *CIAR* Centre for Interdisciplinary Addiction Research, *SIMG* Società Italiana di Medicina Generale, *semFYC* Sociedad Española de Medicina de Familia y Comunitaria. semergen = Sociedad Española de Médicos de Atención Primaria

^a^Refers to number of any response among all contacted individuals

^b^An advertisement in a nationwide newspaper was placed during the period of data collection with very little response. Out of 103 GPs, 98 were from Bavaria and the remaining five from Hesse (1), North Rhine-Westphalia (2) and Saxony (2). The response rate was calculated by omitting the 54,000 potential readers from the denominator

The survey included a number of free text items, including descriptions how alcohol problems were managed. A coding scheme based on free text responses given in Germany and the UK was developed and subsequently all such responses were classified by two independent raters for each language. Kappa agreement coefficients were calculated and ranged from 0.31 to 1 in the variables analyzed. Non-concordant ratings were revisited and a final decision was made by JM.

### Statistical analyses

Three different indicators for good practice alcohol management in patients with HTN were derived from responses given in the questionnaire: a) sufficient screening for alcohol use (at least 7 out of 10 HTN patients); b) sufficient screening (as above) in addition to management of alcohol problems in hypertensive patients with hazardous drinking levels by the GP themselves or within the same practice usually with brief interventions (for rationale see care [21, 29]; c) sufficient screening and management of alcohol dependence in hypertensive patients by the GPs themselves or within the same practice. Indicator c was only met if GPs did not *only* offer brief advice or counselling as management for alcohol dependence but also reported other interventions, such as psychotherapy, or pharmacotherapy. This operationalization was chosen, as current guidelines do not recommend brief advice only as a treatment intervention for dependence [30, 31].

Logistic regressions on each indicator were computed with Stata 14.0 [32], using the following variables as covariates (specifications in parentheses): age (categories as dummy variables with ‘70 or older’ as reference category), sex, country (dummy coded with UK as reference category), beliefs about success of different lifestyle interventions for hypertension (questionnaire items 3 and 4: dummy variables, each scored 1 if rated (highly) successful, else 0), knowledge (questionnaire item 1: dummy variable, scored 1 if alcohol was selected as important risk factor for HTN, else 0), education (questionnaire items 24 and 27: dummy variables, each scored 1 for at least 4 out of 5 points on Likert scale regarding adequacy of graduate education on alcohol/HTN, else 0; questionnaire items 25 and 28: dummy variables, each scored 1 if post-graduate education on alcohol, HTN was received, else 0), and workload (questionnaire item 7: continuous variable containing number of daily patient contacts, z-standardized for each country to achieve comparability). A measure of the respondents’ drinking patterns (questionnaire items 32–34) was also considered for inclusion in the models. However, it was decided against it as it would have overly limited the generalizability of the findings by reducing the sample size by 24 % (from 2468 to 1885) because these items were not assessed among UK respondents and responses were not required to complete the survey in the remaining countries.

## Results

Two thousand four hundred sixty eight health professionals participated in the survey (for details see Table 1).

With respect to the indicators of good practice alcohol management (= main dependent variables), Table 2 gives the prevalence by country.

**Table 2:** Good practice alcohol management by country

	France *N* = 512	Germany *N* = 191	Italy *N* = 360	Spain *N* = 897	UK *N* = 508	Total *N* = 2468
Indicator A:						
Proportion of GPs screening at least 7 out of 10 hypertensive patients for alcohol % (CI)	5.9 (4.1–8.3)	26.7 (20.9–33.4)	36.1 (31.3–41.2)	45.8 (42.6–49.1)	42.5 (38.3–46.9)	34.0 (32.1–35.8)
Indicator B:						
Proportion of GPs with sufficient screening (as A) and self-management of alcohol problems in patients with hazardous drinking levels % (CI)	4.5 (3.0–6.7)	18.8 (13.9–25.0)	26.1 (21.8–30.9)	35.0 (32.0–38.2)	15.7 (12.8–19.2)	22.2 (20.6–23.8)
Indicator C:						
Proportion of GPs with sufficient screening (as A) and self-management of alcohol problems in patients with alcohol dependence ^a^ % (CI)	2.0 (1.0–3.6)	14.1 (9.9–19.8)	3.6 (2.1–6.1)	13.2 (11.1–15.5)	4.7 (3.2–7.0)	7.8 (6.8–8.9)

Notes. *GP* General Practitioner

^a^Treatment of alcohol problems only via brief intervention or advice did not qualify as indicator for sufficient alcohol management

The overview of influencing variables for good practice alcohol management are given in Table 3, where the reference country was always the UK. Clearly screening for alcohol was best implemented in the UK and Spain, management of hazardous drinking levels was best implemented in Spain (87 % of all identified GPs treated only via brief interventions/advice), and treatment of alcohol dependence was best implemented in Spain and Germany. As hypothesized, post-graduate education and the belief that lifestyle interventions were successful in avoiding HTN-related prescriptions seem to impact on all three indicators. For screening and management of hazardous drinking levels, the GPs’ knowledge about the importance of alcohol as a risk factor for HTN was also positively related.

**Table 3:** Prediction of good practice alcohol management

*N* = 2468	Model A^a^		Model B^a^		Model C^a^	
Pseudo R^2^	.1208		.1164		.1113	
Predictors:	OR	*p*	OR	*p*	OR	*p*
Sex: 0 = male, 1 = female	0.80 (0.66–0.97)	.026	1.15 (0.92–1.42)	.221	1.22 (0.88–1.68)	.232
Age: less than 30 years old	0.66 (0.21–2.10)		0.71 (0.20–2.54)		1.20 (0.14–10.57)	
Age: 30–39 years old	0.91 (0.31–2.67)		0.69 (0.21–2.28)		0.97 (0.12–7.81)	
Age: 40–49 years old	0.96 (0.33–2.83)		0.75 (0.23–2.78)		0.84 (0.10–6.77)	
Age: 50–59 years old	0.88 (0.30–2.58)		0.72 (0.22–2.34)		1.19 (0.15–9.42)	
Age: 60–69 years old	0.97 (0.33–2.87)		0.89 (0.27–2.94)		1.10 (0.13–8.88)	
Age: at least 70 years old (reference category^b^)	1	.734	1	.791	1	.714
Country: only France	0.08 (0.05–0.12)		0.20 (0.12–0.32)		0.32 (0.14–0.67)	
Country: only Germany	0.43 (0.29–0.63)		1.04 (0.66–1.65)		2.79 (1.51–5.18)	
Country: only Italy	0.68 (0.49–0.96)		1.48 (0.99–2.21)		0.50 (0.23–1.08)	
Country: only Spain	0.98 (0.76–1.27)		2.57 (1.88–3.51)		2.45 (1.48–4.06)	
Country: UK (reference category^b^)	1	<.001	1	<.001	1	<.001
Belief: Patients successfully reduced blood pressure due to lifestyle change	1.21 (0.96–1.52)	.098	1.18 (0.92–1.51)	.198	1.70 (1.19–2.42)	.003
Belief: Lifestyle changes successful to avoid prescribed HTN medication	1.42 (1.17–1.73)	<.001	1.44 (1.15–1.79)	.001	1.48 (1.07–2.06)	.019
Knowledge: alcohol rated as important risk factor for HTN	1.27 (1.01–1.60)	.043	1.43 (1.10–1.86)	.007	1.21 (0.82–1.79)	.332
Education: university education on alcohol was sufficient	1.41 (1.05–1.90)	.022	1.34 (0.97–1.86)	.079	1.25 (0.78–2.02)	.353
Education: received post-graduate education on alcohol	1.49 (1.23–1.80)	<.001	1.93 (1.55–2.40)	<.001	2.49 (1.75–3.54)	<.001
Education: university education on HTN was sufficient	0.91 (0.75–1.09)	.301	1.04 (0.84–1.29)	.702	0.95 (0.67–1.31)	.752
Education: received post-graduate education on HTN	1.32 (0.98–1.75)	.052	1.32 (0.93–1.89)	.123	1.05 (0.59–1.87)	.865
Workload: country-standardized measure of daily patient contacts	1.02 (0.94–1.12)	.597	1.02 (0.92–1.12)	.718	1.02 (0.90–1.16)	.752

Notes. *HTN* Hypertension

^a^Logistic regression models predicted alcohol management using different indicators: For Model A, sufficient screening, i.e. at least 7 out of 10 HTN patients was predicted. For Model B and C, composite indicators consisting of sufficient screening (as Model A) in addition to self-management of alcohol problems in hypertensive patients with either hazardous drinking levels (Model B) or alcohol dependence (Model C) was predicted. For Model C, treatment of alcohol problems only via brief intervention or advice did not qualify as indicator for sufficient alcohol management

^b^For age and country, the p-values refer to an omnibus test for the entire variable, i.e., testing the global hypotheses that the coefficient for any age category or country deviates from the null hypothesis of no difference above chance

## Discussion

In this large survey, we found that alcohol interventions were relatively scarce in European primary health care. Overall, about one third of the interviewed GPs reported sufficient screening in cases with HTN. One out of five GPs screened and delivered brief interventions in HTN patients with hazardous consumption and about one of 13 GPs provided treatment for HTN patients with alcohol dependence other than advice or brief intervention. There were marked differences between European countries though, with most of the screening and interventions been given in Spain and the UK, and least in France. Compared to British GPs, only a fraction of the French colleagues reported sufficient alcohol screening (OR = 0.08), and only every 50^th^ French GP reported sufficient screening and alcohol management in alcohol dependent patients on their own. We can only speculate about the reasons for the French situation, but it may have to do with lack of guidelines. The French guidelines for HTN treatment developed in 2005 had to be withdrawn in 2011 (http://www.has-sante.fr/portail/jcms/c_272459/fr/prise-en-charge-des-patients-adultes-atteints-d-hypertension-arterielle-essentielle-actualisation-2005-cette-recommandation-est-suspendue) as the authors’ conflict of interest statements did not meet later introduced rules (http://www.has-sante.fr/portail/upload/docs/application/pdf/2011-09/cp_recos_suspendues_19092011_vdef.pdf). In general, the notion of the beneficial effects of alcohol on cardiovascular outcomes is strong (“French paradox”; see [33]; see also the official training materials of the French cardiologists [34]). The lack of knowledge and training in Italian GPs with respect to screening and brief interventions has been found in several other European studies (INEBRIA: AMPHORA: [35]; see also http://www.epicentro.iss.it/alcol/apd2013/presentazioni/9.Cuffari.pdf), and has seemingly not improved over the past years.

Before we discuss potential conclusions of the results, we would like to highlight limitations. First, response rates are relatively low. While it is hard to compare response rates across physicians’ surveys, as there are different sampling frames and several web-based surveys do not even give response rates [36, 37], and even though web-based surveys have comparably lower response rates [38], an overall response rate of 6 % must be considered low. As a consequence, while the national/regional sampling frames can be considered as representative, the low response rates suggest that a convenience sample of GP’s being more motivated and interested in the topic has been drawn [39]. Thus the screening and intervention rates reported are likely to be overestimates (for intervention rates in samples of GP’s with representative sampling and a considerably higher response rate [14, 40]). Second, all answers were self-reports and social desirability bias may have shifted some of our key results upwards [41]. In other words, based on the two major limitations of this study, the rates for screening and interventions among hypertensive primary health care patients in Europe are most likely lower than described in this study. However, given the low response rate, we cannot fully rule out that we have underestimated the GPs’ involvement, e.g., if engaged GPs were too busy to participate in our survey.

## Conclusions

While our findings are susceptible to sample distortion, they are sufficiently robust to demonstrate that the GPs’ involvement in alcohol screening and management among patients with HTN is generally poor in the largest European countries. Thus, the situation for HTN patients is likely not better than for other primary care patients with respect to detection of and interventions for heavy drinking and alcohol use disorders [14, 15, 17, 40]. What can be done about this? First, medical education at universities have to put more emphasis on alcohol as one of the main risk factors for many disease conditions GPs see in their daily practice [40]. The lack of education seems a common problem in all five countries, and was also highlighted in some of the qualitative answers. Moreover, post-graduate training was shown to increase screening and intervention rates [16, 42], and this is, where GP associations can contribute. Secondly, given the high overall workload of GPs, and the overall health burden attributable to alcohol in countries in the European Union [20], alcohol interventions need to be prioritized and this could be done by financial incentives. A recent cluster randomized trial with 746 providers in 120 primary health care centers from five European countries has shown that modest financial incentives increase screening and intervention rates. Interestingly, there is a synergistic effect when financial incentives, training and support are offered together [17]. We hope that the involvement of several GP associations in the current study will help overcome these barriers in the future.

## Additional files

Additional file 1: Structure of the questionnaire. Includes a table of all sections of the questionnaire (DOCX 17 kb) Additional file 2: Questionnaire. Includes the entire questionnaire implemented in the survey (DOCX 35 kb)

## Abbreviations

BASIS: Baseline Alcohol Screening and Intervention Survey
GP: General practitioner
HTN: Hypertension
UK: United Kingdom
