# Supplementary material for: The role of alcohol in the management of hypertension in patients in European primary health care practices – a survey in the largest European Union countries
Source: BMC Fam Pract. 2016 Sep 8;17(1):130. doi: 10.1186/s12875-016-0529-5 (PMC5016945; doi:10.1186/s12875-016-0529-5)
Supplement: Additional file 1: — Structure of the questionnaire. Includes a table of all sections of the questionnaire (DOCX 17 kb) [file 12875_2016_529_MOESM1_ESM.docx]

| **Additional file 1. Structure of the questionnaire** | | |
| --- | --- | --- |
| **Section** | **Brief description** | **N** |
| *Introduction* | - Four small paragraphs on the aims of this study - Mentioning risk factors of HTN but not explicitly alcohol - Disclosure of the study sponsor | 0 |
| *Risk factors of HTN* | - Selection of three out of seven risk factors according to  1. importance (for HTN) and 2. treatability by a general practitioner | 2 |
| *Proposing lifestyle changes* | - Perception of success of advice to change certain lifestyles generally and specific for reduction of alcohol consumption | 4 |
| *HTN in the daily practice* | - Number of daily patient contacts - Number of patients with HTN on an average day - Number of patients with HTN screened for alcohol - Barriers for no or low screening | 4 |
| *Screening for alcohol use* | - Methods/instruments used for screening - Rule followed to screen for alcohol | 2 |
| *Management of HTN and hazardous drinking* | - Steps taken when cases identified | 4 |
| *Management of HTN and alcohol dependence* | - Steps taken when cases identified | 3 |
| *Personal characteristics* | - Sex, age group, profession and specialization | 3 |
| *HTN and alcohol related education* | - Sufficiency of university education, any postgraduate education and perceived competence regarding management of HTN and alcohol | 6 |
| *Blood pressure and AUDIT* | - Blood pressure (systolic and diastolic, not in the UK) - AUDIT-C questionnaire (not in UK) | 5 |
| *Country specific items* | - Region of practice - Mail id for reimbursement or future study participation | 2 |
| *Feedback* | - Free text based question | 1 |
| Note. HTN = Hypertension, AUDIT-C = Alcohol use disorders identification test (short form). N = number of items | | |
