# Supplementary material for: The role of alcohol in the management of hypertension in patients in European primary health care practices – a survey in the largest European Union countries
Source: BMC Fam Pract. 2016 Sep 8;17(1):130. doi: 10.1186/s12875-016-0529-5 (PMC5016945; doi:10.1186/s12875-016-0529-5)
Supplement: Additional file 2: — Questionnaire. Includes the entire questionnaire implemented in the survey (DOCX 35 kb) [file 12875_2016_529_MOESM2_ESM.docx]

## Additional file 2: Questionnaire

**PAGE 1**

# Questionnaire on treatment of lifestyle and hypertension for general practitioners in EU countries

Dear Colleague,

Thank you for participating at this survey on treatment of hypertension in primary health care. The survey will take about 10 minutes to complete and will focus on lifestyle interventions for people with elevated blood pressure.

The current version has some random assignment of lifestyle factors included, but the second part will focus only on one factor as we did not want to take too much of your time.

Thanks a lot for your willingness to participate. The results will inform future treatment of hypertension in primary health care, so your participation is crucial.

If you have any concerns or questions about this questionnaire, please do not hesitate to contact the Principal Investigator of the study, Professor Jürgen Rehm, PhD (via mail [jtrehm@gmail.com](mailto:jtrehm@gmail.com), by indicating in the questionnaire, or via Skype with name “jtrehm1”).

Best wishes

Prof. Jürgen Rehm

TU Dresden, Germany

This survey has been supported financially by H. Lundbeck A/S.

**PAGE 2**

## General questions on treatment of hypertension

1. Please select the three most important risk factors for hypertension.
   - - Lack of **physical activity**
     - Diet with high **salt intake**
     - **Alcohol use**
     - **Smoking**
     - Overweight and **obesity**
     - **Stress**
     - **Sleep apnoea**

**PAGE 3**

## General questions on treatment of hypertension

1. Thinking of your patients, which of the risk factors for hypertension could be most easily dealt with by a GP?

Pick two risk factors that are easiest to address for GPs:

- Lack of **physical activity**
  - - Diet with high **salt intake**
    - **Alcohol use**
    - **Smoking**
    - Overweight and **obesity**
    - **Stress**
    - **Sleep apnoea**

**PAGE 4**

## General questions on treatment of hypertension

1. Looking at your experience, how successful have your hypertensive patients been **in reducing their blood pressure** when you asked them to make lifestyle changes?

- highly successful
- successful
- moderately successful
- not at all successful

1. In your view, how successful are lifestyle changes **to avoid prescribing medication for hypertension**?
   - highly successful
   - successful
   - moderately successful
   - not at all successful
2. Think about the last 10 patients you have seen with hypertension, how many have avoided medication by changing their lifestyle following an interventions in your clinical practice?

Please enter a number between 0 and 10 below: ___

**The next questions are specific to one lifestyle factor, alcohol consumption, with respect to hypertension.**

1. In your view, how many of these cases (avoiding prescription of medication for hypertension) are related to a reduction in alcohol consumption?

Please enter a number between 0 and 10 below (the number given here must be smaller than the number given in the previous question): ___

**PAGE 5**

## Screening and Diagnosis

1. How many patient contacts do you have personally on an average day in your practice?

***____*** [error message: Please enter a number between 1 and 999]

1. How many of these patients have hypertension?

***____***[ error message: Please enter a number between 1 and 999]

1. In how many out of 10 patients with hypertension, i.e. of the patients you indicated in question 8, did you ever screen for **alcohol use**, **hazardous drinking** or **alcohol dependence**?

***____***

1. If answer to add is smaller or equal to 3: What are the main barriers to screening for alcohol use, hazardous drinking or alcohol dependence?

***____________________________________*** [open question]

**PAGE 6**

[only viewed if at least one screening was reported in item 9]

## Screening and Diagnosis

1. How did you screen for **alcohol use** or **hazardous drinking** (multiple answers possible)?
   - AUDIT
   - AUDIT-C
   - Asked in my own words
   - Other, please specify: ___________
2. When have you screened for **alcohol use** or **hazardous drinking** in patients with hypertension (multiple answers possible)?

- In patients with newly detected hypertension
- In patients with hypertension if their blood pressure was very high
- In patients with treatment resistant hypertension
- Other***,*** please specify: ______*__*

**PAGE 7**

## Access and Pathways

1. What actions do you take when you diagnose someone with both **hazardous drinking levels** and **hypertension**?

- Manage both conditions/problems myself
- Both conditions/problems will be managed within our practice by another member of the primary care team
- Manage only hypertension
- Manage only hypertension and refer to another service for hazardous drinking intervention
- Other, please specify:

**PAGE 8**

[only viewed if answered a in item 13]

## Access and Pathways

1. You reported managing both **hazardous drinking levels** and **hypertension** yourself. What interventions did you use for hazardous drinking?
   - - Advice
     - Brief interventions
     - Other, please specify:

**PAGE 9**

[only viewed if answered b in item 13]

## Access and Pathways

1. You reported that both **hazardous drinking levels** and **hypertension** are managed within your practice. What interventions are done for hazardous drinking?
   - - Advice
     - Brief interventions
     - Other, please specify:

**PAGE 10**

[only viewed if answered c in item 13]

## Access and Pathways

1. You reported that you manage only hypertension in patients with both **hazardous drinking levels** and **hypertension**. Please specify reasons why you manage only hypertension in these patients?

______________________________________________ [open question]

**PAGE 11**

## Access and Pathways

1. What actions do you take when you diagnose someone with both **alcohol dependence** and **hypertension**?

- Manage & treat both diseases/conditions myself
- Manage & treat both diseases/conditions within our practice
- Manage only hypertension and refer to other service for alcohol dependence intervention
- Manage only hypertension and refer to specialist care for alcohol dependence
- Other, please specify: ______________

**PAGE 12**

[only viewed if answered a in item 17]

## Access and Pathways

1. You reported managing both **alcohol dependence** and **hypertension** yourself. How do you treat alcohol dependence yourself?

_____________________________________________________

**PAGE 13**

[only viewed if answered b in item 17]

## Access and Pathways

1. You reported that both **alcohol dependence** and **hypertension** are managed within your practice. How is alcohol dependence treated in your practice?

_____________________________________________________

**PAGE 14**

## Personal ch**a**racteristics of the respondent

1. Sex:
   - man
   - woman
2. Age:

- < 30 years
- 30-39 years
- 40-49 years
- 50-59 years
- 60-69 years
- > 69 years

1. Profession and specialisation [country-specific categories shall be used here]:

- GP
- Consultant Physician
- Consultant Cardiologist
- Consultant (other)
- Other profession, please specify: ______________

**PAGE 15**

[Only displayed if question 22 has been answered “Consultant (other)”]

## Personal ch**a**racteristics of the respondent

1. Please specify type of other specialist: _______________

**PAGE 16**

## Personal ch**a**racteristics of the respondent

1. Did you find your education at university dealt sufficiently with the topics of **alcohol, alcohol use disorders and health**?

The topic was covered …

[5 POINT SCALE:] not at all --- very sufficiently

1. Did you have any postgraduate training for dealing with hazardous drinking or alcohol dependence?

- No
- Yes

1. Do you feel competent dealing with patients who have **hazardous drinking levels** or **alcohol dependence**?

- I do not feel competent dealing with neither hazardous drinking nor alcohol dependence
- I do not feel competent dealing with hazardous drinking, but with alcohol dependence
- I do not feel competent dealing with alcohol dependence but with hazardous drinking
- I feel competent dealing with both hazardous drinking and alcohol dependence

1. Did you find your education at university dealt sufficiently with the topic of **blood pressure and hypertension**?

The topic was covered …

[5 POINT SCALE:] not at all --- very sufficiently

1. Did you have any postgraduate training for dealing with hypertension?

- No
- Yes

1. Do you feel competent dealing with patients who have hypertension?

- No
- Yes

**PAGE 17 [not assessed in the UK]**

## Personal ch**a**racteristics of the respondent

Lastly, we are also interested in your own blood pressure and drinking behavior. The following questions are voluntarily and strictly confidential.

Please enter your current blood pressure (in mm Hg) below:

1. Systolic:

_ _ _ [error message: Please enter a number between 10 and 299.]

1. Diastolic:

_ _ _ [error message: Please enter a number between 10 and 299.]

1. [AUDIT-C] How often do you have a drink containing alcohol?
   1. Never
   2. Monthly or less
   3. 2-4 times a month
   4. 2-3 times a week
   5. 4 or more times a week
2. [AUDIT-C] How many standard drinks containing alcohol do you have on a typical day?
   1. 1 or 2
   2. 3 or 4
   3. 5 or 6
   4. 7 to 9
   5. 10 or more
3. [AUDIT-C] How often do you have six or more drinks on one occasion?
   1. Never
   2. Less than monthly
   3. Monthly
   4. Weekly
   5. Daily or almost daily

**LAST PAGE**

## End of the survey

1. Thank you for participating at this survey.
   If you would like to give any feedback, please use the text box below:

|  |
| --- |
